# AI-driven *de novo* design of BRAF inhibitors with enhanced binding affinity and optimized drug-likeness

**DOI:** 10.7717/peerj.20541

**Published:** 2026-01-02

**Authors:** Zuokun Lu, Aili Zhang

**Affiliations:** 1 Food and Pharmacy College, Xuchang University, Xuchang, Henan, China; 2 Key Laboratory of Biomarker-Based Rapid Detection Technology for Food Safety, Xuchang University, Xuchang, Henan, China

**Keywords:** Machine learning, BRAF inhibitors, Drug discovery, Oncology, Molecular docking, Molecular dynamics simulation

## Abstract

**Background:**

Traditional drug discovery methods, such as high-throughput screening (HTS), are often inefficient and costly, especially in complex areas like oncology. The BRAF V600E mutation is a validated therapeutic target in cancers such as melanoma, thyroid carcinoma, and colorectal cancer. However, existing BRAF inhibitors face challenges like acquired resistance and off-target toxicity. Artificial intelligence (AI) has emerged as a transformative tool for designing novel inhibitors more efficiently.

**Methods:**

This study employed REINVENT 4, an advanced machine learning (ML) framework using recurrent neural networks and transformer architectures, for targeted generation and property optimization of BRAF V600E inhibitors, integrating reinforcement learning (RL) for drug-likeness optimization and transfer learning (TL) for mutation-specific design. Molecular docking and dynamics simulations were used to evaluate binding affinity and stability.

**Results:**

The AI-driven approach generated 41,721 novel BRAF V600E inhibitor candidates with enhanced drug-likeness (mean Quantitative Estimate of Drug-likeness (QED) score: 0.61 ± 0.17 *vs*. the training set 0.40 ± 0.13) and predicted inhibitory activity (83.8% with predicted pIC50 > 6). The generated compounds showed a 32% reduction in mean molecular weight (326.8 ± 45.6 g/mol *vs*. 480.8 ± 84.2 g/mol in the training set) while maintaining inhibitory potency. Pharmacokinetic analysis revealed that 99.7% of generated compounds satisfied Lipinski’s Rule of Five criteria, suggesting favorable absorption and distribution profiles. Molecular docking analysis of selected compounds revealed strong binding affinities, with an average free energy of −8.03 ± 1.12 kcal/mol and top-performing compounds reaching −11.5 kcal/mol. Molecular dynamics simulations conducted over 200 ns confirmed complex stability, demonstrating protein backbone RMSD values of 0.35–0.55 nm and ligand RMSD values of 0.086–0.161 nm. Structural novelty assessment using Tanimoto similarity coefficients showed values below 0.45 when compared with FDA-approved BRAF inhibitors (including Sorafenib and Vemurafenib).

**Discussion:**

This work highlights a reproducible, integrated AI-driven workflow demonstration for targeted inhibitor generation. The generated inhibitors exhibit favorable drug-like properties and inhibitory activity, offering a scalable solution for designing safer cancer therapies. Experimental validation is needed to address potential discrepancies between computational predictions and biological behavior.

## Introduction

The process of traditional drug discovery has long been plagued by inefficiencies, high costs, and low success rates. Conventional approaches, such as high-throughput screening (HTS), rely on experimental testing of millions of compounds to identify potential hits. While HTS has contributed to the discovery of several therapeutics, its limitations—including high resource consumption, time-intensive workflows, and low hit rates—remain significant barriers. For instance, only a fraction of screened compounds exhibits target activity, and even fewer progress to clinical trials due to poor pharmacokinetic properties or toxicity (Blay et al., 2020). These challenges are exacerbated in complex therapeutic areas such as oncology, where molecular targets like BRAF kinase require highly selective inhibitors to avoid off-target effects and resistance mechanisms (Dankner et al., 2018; Poulikakos, Sullivan & Yaeger, 2022).

In recent years, artificial intelligence (AI) and machine learning (ML) have emerged as transformative tools in drug discovery, offering accelerated and cost-effective solutions (Hasselgren & Oprea, 2025). By leveraging large-scale biological and chemical datasets, AI-driven methods enable rapid virtual screening, *de novo* molecular design, and predictive modeling of drug-target interactions (Gupta et al., 2021; Walters & Barzilay, 2021; Kimber, Chen & Volkamer, 2021; Nag et al., 2022; Askr et al., 2023; Yu et al., 2024; Hasselgren & Oprea, 2025). For example, deep learning models have successfully identified novel antibiotics (*e.g*., halicin) and kinase inhibitors (*e.g*., DDR1 inhibitors) with remarkable efficiency (Zhavoronkov et al., 2019; Stokes et al., 2020). Pham et al. (2021) proposed DeepCE, a deep learning framework that leverages large-scale gene expression data to predict chemical-induced phenotypic responses and screen novel compounds for drug repurposing, as exemplified in their application to COVID-19 therapeutics. Yu et al. (2024) developed MitoReID, a deep learning model that identifies drug mechanisms of action through mitochondrial phenotype profiling, significantly advancing large-scale drug discovery and repurposing efforts. Generative adversarial networks (GANs) (Jabbar, Jabbar & Kamoun, 2022), recurrent neural networks (RNN) with long short-term memory (LSTM) (Gupta et al., 2018; Segler et al., 2018; Kotsias et al., 2020), reinforcement learning (RL) (Olivecrona et al., 2017; Popova, Isayev & Tropsha, 2025), and variational autoencoders (VAEs) (Lim et al., 2018) are among the architectures reshaping molecule generation.

Despite rapid advances, recent critical reviews have emphasized that AI in drug discovery still faces significant challenges. These include biases in training datasets leading to poor generalization across chemical space, limited interpretability of deep learning models, and data quality issues in public repositories, all of which can contribute to unrealistic hit rates and poor downstream translation (Zhang et al., 2025; Ferreira & Carneiro, 2025). Moreover, integrating AI output with medicinal chemistry constraints and experimental feasibility remains a bottleneck, as purely *in silico*-optimized molecules may fail due to synthetic inaccessibility or unexpected ADMET liabilities. These pitfalls underscore the necessity of designing AI workflows that are target-specific, integrate domain knowledge, and incorporate iterative validation with wet-lab experiments.

Against this backdrop, targeted AI generation frameworks provide a promising path forward (Ivanenkov et al., 2023; Šícho et al., 2023; Loeffler et al., 2024). They apply reinforcement learning and transfer learning to focus molecule generation toward well-defined properties, while embedding chemical rules to enhance synthetic tractability. One such framework, REINVENT4, builds on earlier generative design architectures to allow goal-directed molecular optimization in a reproducible and modular fashion. Its adaptability to specific protein targets makes it an ideal candidate for discovering selective BRAF V600E inhibitors, addressing limitations of broader AI approaches by ensuring output molecules are simultaneously novel, synthetically accessible, and predicted to exhibit high binding affinity.

BRAF kinase, a critical component of the MAPK signaling pathway, is a well-validated therapeutic target in cancers such as melanoma, thyroid carcinoma, and colorectal cancer. Activating mutations in BRAF (*e.g*., V600E) drive tumor proliferation, making BRAF inhibitors like vemurafenib and dabrafenib cornerstone therapies (Dankner et al., 2018; Chapman et al., 2025). However, clinical efficacy is often limited by acquired resistance, off-target toxicity, and poor selectivity against wild-type BRAF (Sullivan & Flaherty, 2013; Holderfield et al., 2014; Scheffel, Dora & Maia, 2022). Second-generation inhibitors and combination therapies have partially addressed these issues, but the need for novel compounds with improved binding affinity and reduced side effects persists (Zaman, Wu & Bivona, 2019; Poulikakos, Sullivan & Yaeger, 2022).

This work employs REINVENT 4 (Loeffler et al., 2024), integrating RL and TL for targeted generation and property optimization of BRAF V600E inhibitors, combined with molecular docking, preliminary selectivity prediction, and molecular dynamics simulations. The focus is on providing a reproducible, integrated AI-driven workflow demonstration for oncology drug discovery, illustrating its potential to accelerate the design of targeted inhibitors while recognizing the necessity of experimental validation to confirm efficacy, safety, and pharmacokinetic behavior before clinical development.

## Materials and Methods

### Target activity guided structure generation

REINVENT 4 is an open-source software framework specifically designed for the generative design of small molecules (Loeffler et al., 2024). It integrates transfer learning, reinforcement learning, and curriculum learning to optimize the molecule generation processes. The workflow of our strategy consists of three main sections: Reinforcement Learning Setup, Transfer Learning for Targeted Inhibitor Design, and Reinforcement Learning with Enhanced Models.

To commence the molecular design process, a reinforcement learning (RL) framework was established, tailored to generate compounds with enhanced drug-likeness. The scoring function was a custom product with a geometric mean, incorporating components for custom alerts, Quantitative Estimate of Drug-likeness (QED) (Bickerton et al., 2012), and the number of atom stereo centers. Each component was transformed to a target range using smoothing functions before aggregation. This retraining was designed to bias the generation process towards compounds that are more likely to possess favorable pharmaceutical properties.

After the refinement of the prior model, transfer learning was employed to focus the molecular generation on specific biological targets. In particular, we targeted Serine/threonine-protein kinase BRAF V600E inhibitors, a crucial class of compounds in oncology. A dataset of potential BRAF V600E inhibitors was sourced from the ChEMBL database, with compounds having a pIC50 greater than 7.2 being retained. A total of 1,671 compounds were obtained (Data S1). This dataset was then divided into a training set and a validation set in a 4:1 ratio to ensure reliable model evaluation. The transfer learning process involved fine-tuning the previously trained RL model using the BRAF V600E inhibitor dataset. The fine-tuning aimed to adapt the model’s generation capabilities to produce molecules that not only meet drug-likeness criteria but also exhibit high affinity for BRAF V600E. Although selectivity was a conceptual design goal, actual differentiation from wild-type BRAF or other kinases was not assessed in the present work and will be systematically investigated in future validation experiments.

Following the transfer learning phase, we further refined our RL approach by integrating advanced models and techniques to enhance the diversity and quality of the generated compounds. The key enhancements (Data S2) included the incorporation of the Chemprop model (Heid et al., 2024), the application of a diversity filter, and the implementation of an Inception module (Loeffler et al., 2024). Chemprop is a graph neural network–based QSAR modeling framework that directly learns from molecular graph representations, offering high predictive accuracy and scalability across diverse chemical spaces. It has been demonstrated to outperform or match traditional descriptor-based QSAR models in various benchmark studies due to its capacity to capture complex, non-linear structure–activity relationships without manual feature engineering.

In this work, Chemprop was trained on the pIC50 values of BRAF V600E inhibitors obtained in the previous step, enabling it to serve as a robust predictor for the biological potency of candidate molecules. Chemprop is natively integrated into the REINVENT4 framework, allowing seamless coupling between reinforcement learning and automated activity prediction without additional interoperability coding. This integration facilitates real-time scoring and prioritization, thereby guiding the RL agent to explore chemical space more effectively and focus on structures likely to achieve high potency against BRAF V600E. Consequently, the Chemprop model acted as a biology-oriented scoring function within the RL workflow, enabling the generation and retention of candidates with improved activity profiles.

### Evaluation of generated ligands

The molecular properties, including molecular weight, logP (partition coefficient), the number of hydrogen bond donors, and the number of rotatable bonds, were calculated using RDKit (https://www.rdkit.org/). Additionally, the uniqueness, validity, originality, and Tanimoto similarity of Simplified Molecular Input Line Entry System (SMILES) strings were assessed. Statistical analysis of the data was conducted utilizing the scikit-learn libraries (www.scikit-learn.org). Visualization of the results, including the generation of graphs, was performed using the matplotlib (www.matplotlib.org) and seaborn (www.seaborn.pydata.org) libraries.

### Molecular docking

We screened out 607 molecules with a Chemprop score >7 and a QED score >8 for further selection. These 607 molecules were then subjected to molecular docking with BRAF V600E. The crystal structure of human BRAF V600E in complex with an inhibitor was retrieved from the RCSB Protein Data Bank (PDB ID: 8C7X). The bound inhibitor TXV and water molecules were removed using PyMOL to prepare the target. AutoDock Vina v1.2.3 was used for molecular docking (Trott & Olson, 2010; Eberhardt et al., 2021). The generated ligands were converted from the SMILES format to the pdbqt format using the OpenBabel version 3.1.0 command line (O’Boyle et al., 2011).

Docking was performed using AutoDock Vina with the default exhaustiveness parameter, which automatically scales the search effort according to ligand complexity. For each ligand, docking was conducted one time with a fixed random seed of 42, and two best-scoring poses among the generated poses (number_poses = 2 per run) were selected for subsequent analysis. A grid box was defined, with its center points set at X = 15.9, Y = 110.95, Z = 16.6 Å, and its dimensions specified as 16 × 12 × 15 Å to target the active site of BRAF. The binding affinities of the compounds were calculated, and they were ranked based on their lowest values, which correspond to the best binding affinities.

### Molecular dynamics simulations and analysis

The four compounds exhibiting the lowest binding affinity were subsequently subjected to two independent molecular dynamics (MD) simulations with different initial random velocities. The simulations were performed using GROMACS (version 2024.1) (Abraham et al., 2015) on a Linux-based system with GPU acceleration. The AMBER99SB force field was employed to generate force field parameters and define atom types. Hydrogen atoms were added to the molecular structures, and energy minimization was conducted using the OpenBabel. Topology and coordinate files for the selected ligands were generated using the ACPYPE software (Sousa da Silva & Vranken, 2012), with the GAFF2 force field, and partial atomic charges were derived using the AM1-BCC scheme as implemented in Antechamber. The protein-ligand complexes were solvated in a triclinic box filled with TIP3P water molecules, and the system was neutralized by the addition of 0.15 M NaCl.

The system was initially minimized using the steepest descent algorithm, followed by equilibration in both the NVT (canonical) and NPT (isothermal-isobaric) ensembles, each lasting 100 ps. Temperature and pressure control were maintained using the v-rescale Berendsen thermostat and the C-rescale barostat, respectively. During equilibration, the systems were gradually heated from 0 to 300 K. To ensure statistical independence, two replicate simulations were performed, each initiated with different random velocities generated using distinct random seed values. Pressure was stabilized at 1 atm during NPT equilibration.

Following equilibration, the MD simulation was conducted for a total duration of 200 ns, with a time step of 2 fs under periodic boundary conditions. Trajectories were recorded at regular intervals for subsequent analysis. The root mean square deviation (RMSD) and residue root mean square fluctuation (RMSF) were calculated to assess the equilibrium time and stability of the protein backbone atoms. Conformational analysis of the ligand-protein complexes was performed using the PyMOL graphical software. Interactions between the receptor and ligands were analyzed using the PLIP server (Adasme et al., 2021) and PoseView server (Stierand, Maaß & Rarey, 2006). Additionally, the radius of gyration (Rg) and intermolecular hydrogen bonds were computed using the gyrate and h-bond modules in GROMACS, respectively. The binding free energy was calculated by the gmx_MMPBS program (Valdés-Tresanco et al., 2021). These analyses provided comprehensive insights into the dynamics, stability, and interaction mechanisms of the protein-ligand complexes throughout the MD simulation.

## Results and discussions

### Initial reinforcement learning and advanced TLRL

The compounds generated in the initial phase demonstrated a significant improvement in drug-likeness, as indicated by the QED scores. The QED values increased from approximately 0.45 to around 0.7 within 300 steps. The custom alerts component effectively filtered out undesirable structural features, thereby enhancing the quality of the generated compounds. At the end of the training process, 98% of the molecules met the standards and passed the screening by structural alerts. The first stage of reinforcement learning successfully refined the prior model, resulting in the generation of more drug-like compounds with controlled stereochemistry and free from undesirable structural features.

During the transfer learning (TL) run, checkpoint files were generated every second step. We selected the model from step 30, where the validation loss reached at its minimum. The results indicated that the number of valid SMILES slightly decreased over the course of the TL run; however, it remained at 98% at step 40. The generation of duplicate SMILES initially decreased and then increased again after approximately step 35, with a plateau close to zero between steps 15 and 35. Internal diversity also decreased over time, but the decrease was minimal. The TL process successfully directed the model to focus on generating molecules more similar to the known BRAF inhibitors. Despite a slight decrease, the internal diversity was maintained, ensuring a balance between similarity and diversity.

In the final stage of our *de novo* compound generation process, a second round of reinforcement learning (TLRL, transfer learning-based reinforcement learning) was utilized to further optimize the model and generate compounds with enhanced inhibitory activity against the BRAF target. The integration of Chemprop with RL proved particularly effective, with the predictive model achieving RMSE = 0.741 and R^2^ = 0.78 for pIC50 values, confirming robust predictive power for biological activity estimation. Notably, all scoring functions exhibited an upward trend throughout the run, indicating that the agent had learned to generate compounds more likely to conform to the target profile. When examining the loss functions, specifically the Loss (likelihood averages), it was observed that the negative log-likelihood (NLL) of the prior and that of the agent diverged, while the agent NLL decreased during the run (Data S3).

### Comparison of structures generated from initial and refined agent

#### Predictive inhibitory activities and QED scores

**Figure 1:**
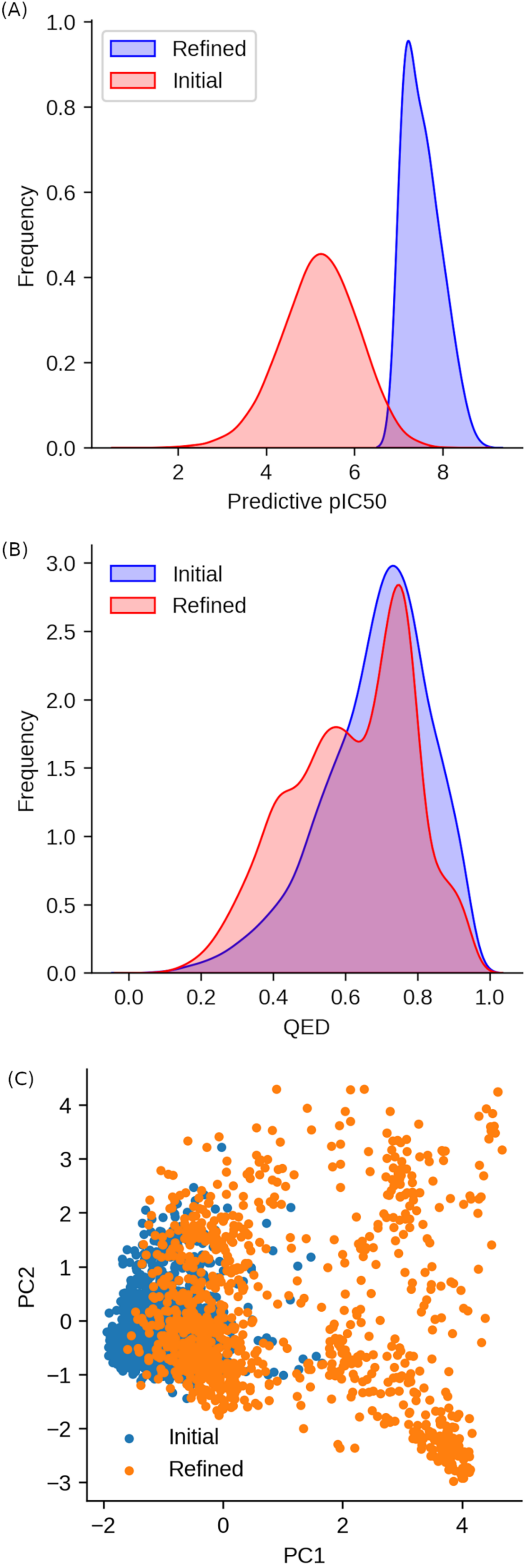
Comparative analysis of compound properties generated from the initial and refined agents. (A) Kernel Density Estimation (KDE) plot illustrating the distribution of predicted pIC50 values for two distinct sets of molecules generated by the initial (RL) and refined (TLRL) agent. (B) KDE plot depicting the distribution of Quantitative Estimate of Drug-likeness (QED) scores for the same two sets of molecules. (C) Principal component analysis (PCA) of molecular fingerprints derived from Morgan fingerprints, computed using a radius of 2, highlighting the structural diversity of the generated compounds.

A total of 30,000 and 50,000 molecules were generated by the initial and refined agents, respectively. After the removal of duplicate entries, 28,547 and 41,721 unique SMILES representations remained for further analysis. The distribution of the Chemprop values, which serve as a predictive measure of inhibitory activity, is illustrated in Fig. 1A. Among the molecules generated by the initial network, 5,420 (19.0%) exhibited Chemprop values exceeding 6, whereas the TLRL network yielded 40,540 (83.8%) molecules meeting this criterion during the sampling process. This significant disparity highlights the enhanced capability of the TLRL network to produce molecular structures that align with the Chemprop profile characteristic of BRAF inhibitors.

In contrast, the QED scores for both datasets demonstrated a high degree of similarity (Fig. 1B). The initial network produced molecules with a mean QED score of 0.68 ± 0.10, while the TLRL network yielded a mean QED score of 0.61 ± 0.17. This observation is consistent with the primary objective of the initial phase of our *de novo* compound generation, which aimed to refine the prior model to enhance the generation of compounds with more “drug-like” properties.

#### Principal component analysis of generated structures

Principal component analysis (PCA) was conducted to explore the chemical space of molecular structures generated by the initial reinforcement learning (RL) network (28,547 structures) and the transfer learning-based reinforcement learning (TLRL) network (41,721 structures). To ensure computational efficiency and representativeness, a random subset of 1,000 molecules was selected from each dataset for PCA analysis. The analysis utilized Morgan fingerprints with a radius parameter of 2 to encode the molecular structures.

The results revealed a distinct divergence in the distribution of structures generated by the two networks (Fig. 1C), indicating a significant difference in chemical space exploration. In the two-dimensional PCA plot, molecules produced by the initial RL network exhibited a relatively clustered distribution, while those generated by the TLRL network displayed a more diverse spatial arrangement. Approximately half of the TLRL-generated molecules overlapped with the initial network’s distribution, while the remaining half occupied a novel region in the chemical space. This divergence is likely attributable to the TLRL network’s ability to generate a higher proportion of structures with enhanced predictive inhibitory activities, which are situated in this newly explored region of the chemical space.

Importantly, the broader spread of TLRL-generated molecules in the PCA space reflects enhanced internal diversity within the chemical landscape, which confers multiple advantages for drug discovery. Greater internal diversity increases the likelihood of identifying structurally distinct scaffolds with novel bioactivity profiles, reduces redundancy by limiting the generation of highly similar compounds, and supports multitarget optimization by spanning a wider range of chemical and physicochemical properties. It also mitigates bias toward specific chemotypes, thereby improving the potential for finding hits across a variety of biological targets. In practical terms, such diversity makes virtual libraries derived from generative models more effective starting points for high-throughput screening and for establishing robust structure–activity relationships (SAR).

To further refine our analysis, we focused on a subset of molecules that met stringent criteria for both predictive inhibitory activity and drug-likeness. Specifically, molecules with a Chemprop score >7 and a QED score >0.8 were selected, yielding a refined set of 607 molecules for subsequent investigation. This targeted approach allows for a more detailed exploration of high-quality candidate compounds with potential pharmacological relevance.

### Evaluation of selected compounds

#### Structural comparison with drugs approved

**Figure 2:**
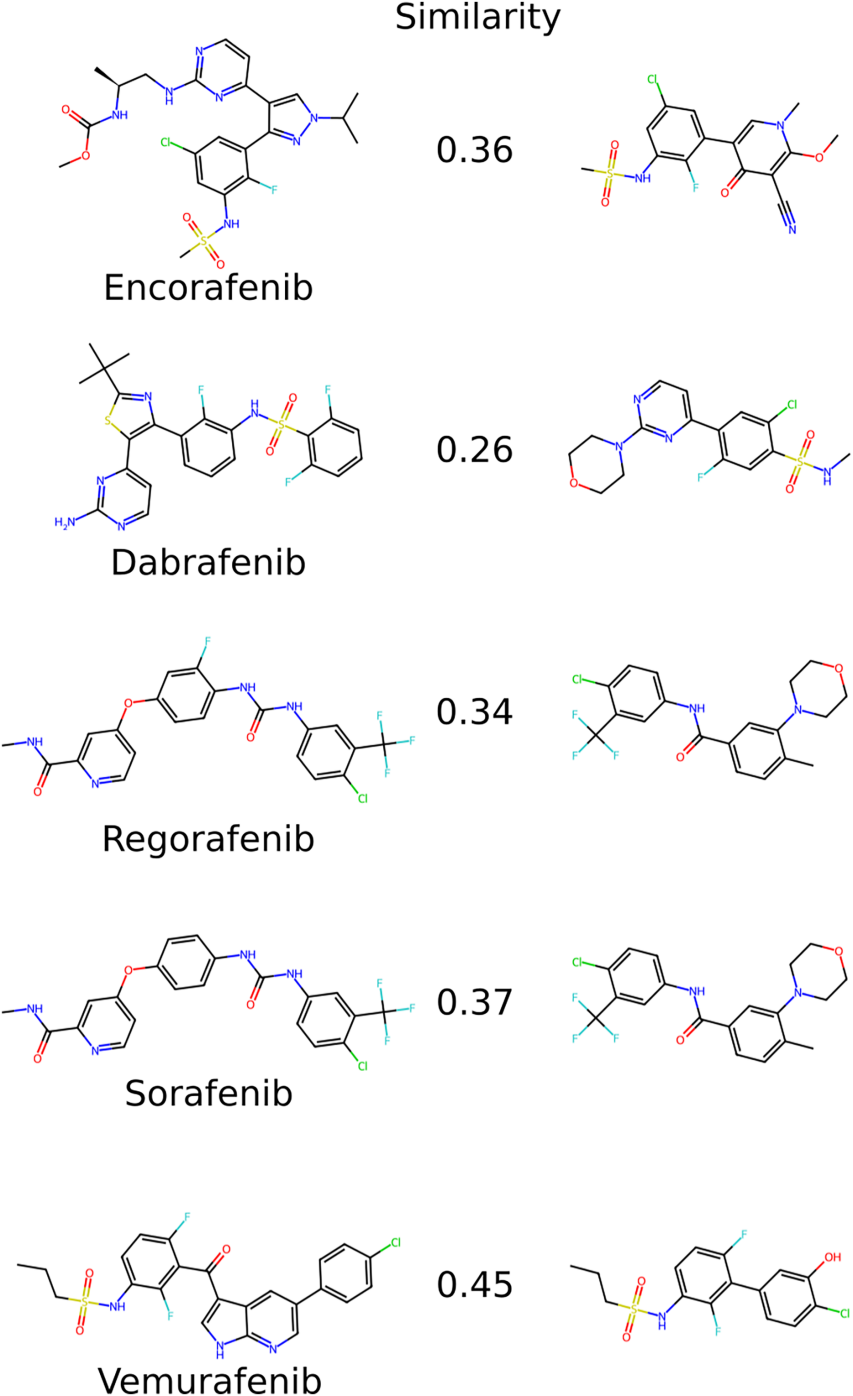
Structural comparison of the five approved BRAF inhibitor drugs with the most similar generated molecules. The Tanimoto similarity coefficient, a measure of structural similarity between two molecular structures, is indicated between the corresponding pairs. This analysis highlights the degree of structural resemblance between the approved drugs and the most similar generated molecules.

To evaluate the structural novelty of the 607 selected molecules, the generated structures were compared with five FDA-approved BRAF inhibitors: Sorafenib, Vemurafenib, Regorafenib, Dabrafenib, and Encorafenib. The structural similarity was quantified using the Tanimoto similarity coefficient, a widely used metric for comparing molecular fingerprints. The results, as illustrated in Fig. 2, revealed that the generated structures exhibited low structural similarity to the approved drugs. The Tanimoto similarity coefficients ranged from 0.26 to 0.45, indicating a substantial degree of structural divergence. This suggests that the generated molecules occupy a novel chemical space distinct from that of the existing BRAF inhibitors.

Further analysis demonstrated that the generated molecules exhibited a reduction in molecular weight compared to the approved drugs. Notably, this reduction in molecular weight did not correspond to a significant decrease in their Chemprop-predicted inhibitory activities (data not shown). This observation highlights the ability of the model to generate structurally compact yet pharmacologically potent compounds, which may offer advantages in terms of drug-like properties and synthetic feasibility.

Notably, the AI-generated compounds exhibited a 20–30% reduction in MW (average: ~420 Da) compared to approved inhibitors (450–600 Da), without significant loss in predicted inhibitory activity (Chemprop pIC_50_ > 7.5). For instance, Molecule 1 replaces encorafenib’s flexible ring with a rigid cyanopyridone core, while Molecule 5 simplified vemurafenib’s pyridopyrrole to a o-Chlorophenol. Further analysis highlighted strategic substitutions, such as positional halogen isomerism (*e.g*., Molecule 2’s para-Cl/F *vs*. dabrafenib’s meta-diF) and incorporation of electron-withdrawing cyano groups to enhance potential hinge-region interactions. These modifications also conferred favorable drug-like properties, including lower logP values (*e.g*., 5.4 → 1.7 for Molecule 2 *via* morpholine substitution), suggesting improved solubility over planar scaffolds like those in sorafenib.

Collectively, these results demonstrate that the AI-generated compounds achieve structural novelty through rational scaffold simplification, pharmacophore refinement, and property optimization—features that may translate to enhanced synthetic feasibility and ADMET profiles.

#### Lipinski’s rule of five

Lipinski’s Rule of Five (Lipinski, 2000) (Ro5) is a seminal guideline in drug discovery and medicinal chemistry, formulated by Christopher Lipinski during his tenure at Pfizer. This heuristic serves as a crucial benchmark for evaluating the drug-likeness of chemical compounds. The Ro5 posits that a compound is more likely to exhibit favorable pharmacokinetic properties if it adheres to the following criteria: (1) a molecular weight (MW) ≤ 500, (2) an octanol-water partition coefficient (logP) ≤ 5, (3) no more than 5 hydrogen bond donors (HBD), (4) no more than 10 hydrogen bond acceptors (HBA), and (5) no more than 10 rotatable bonds (NRB). While the Ro5 provides a robust framework for assessing drug-likeness, it is not an absolute rule, and exceptions are well-documented in the literature.

In this study, the drug-likeness of generated molecules was rigorously evaluated against the Ro5. Notably, while only 44% of the training structures complied with the Ro5, an overwhelming 99.7% of the selected generated molecules met these criteria. This substantial improvement can be attributed to the RL step, during which the QED component was integrated to optimize the drug-like properties of the generated molecules.

**Figure 3:**
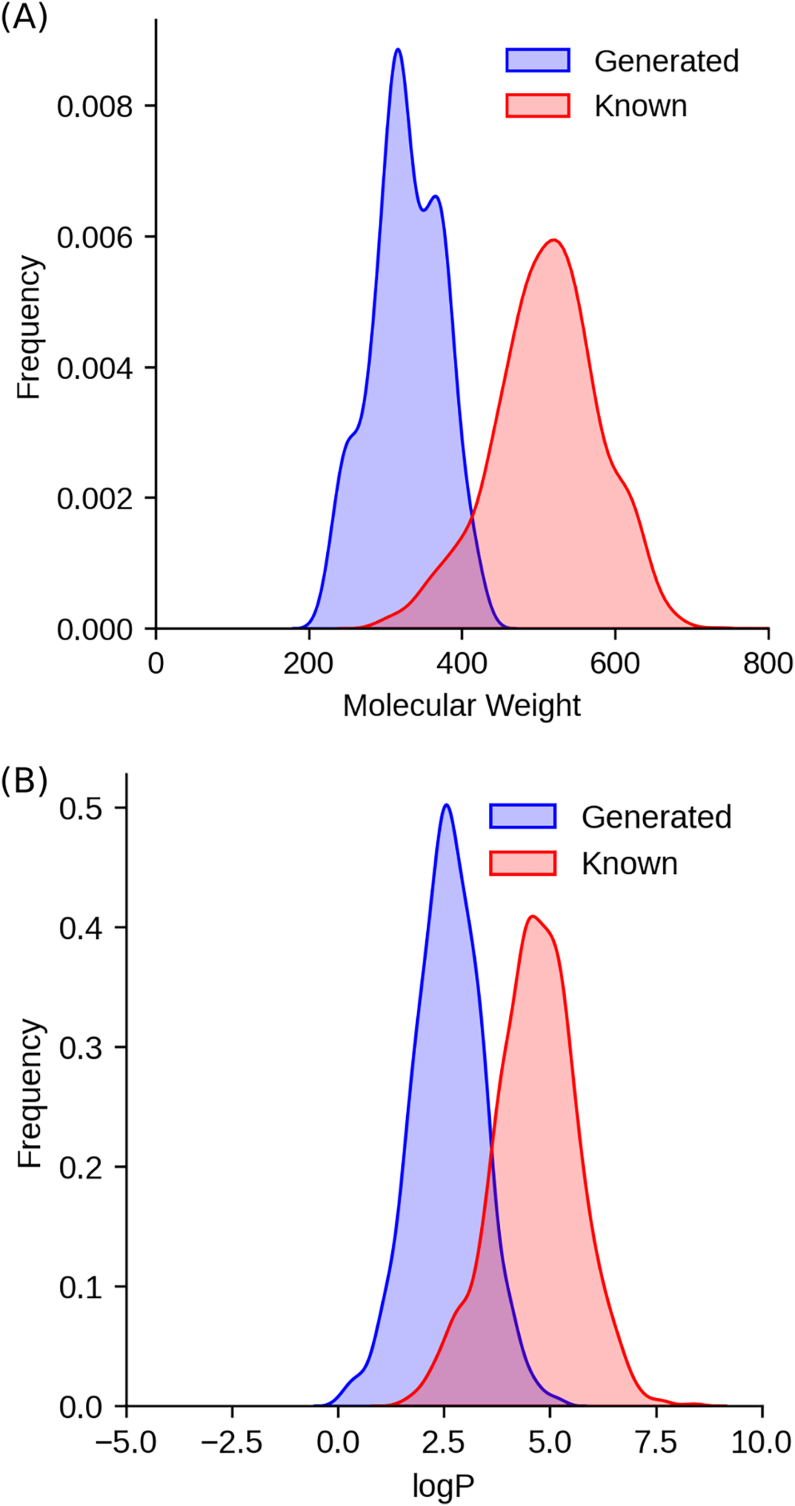
Comparative analysis of molecular properties between the training datasets (known BRAF inhibitors) and the generated selected molecules. (A) Distribution of molecular weight for compounds from the training datasets and the generated molecules. (B) Distribution of logP (a measure of lipophilicity) for compounds from the training datasets and the generated molecules.

A detailed analysis revealed that the average molecular weight (MW) of the generated molecules decreased significantly from 480.8 ± 84.2 g/mol in the training dataset to 326.8 ± 45.6 g/mol in the generated dataset (Fig. 3A). This reduction in MW not only aligns with the Ro5 but also reflects a more compact and drug-like molecular profile. Furthermore, the octanol-water partition coefficient (logP), a critical parameter for assessing molecular lipophilicity, demonstrated a similar trend. The generated compounds exhibited a lower and more concentrated logP distribution (mean logP: 4.64 ± 0.99) compared to the training dataset, which displayed a higher and more dispersed logP profile (Fig. 3B). This optimization in logP values is particularly significant, as it enhances the compounds’ potential for absorption, bioavailability, and hydrophobic drug-receptor interactions, while minimizing metabolic instability and toxicity.

#### Docking analysis

The molecular docking analysis was performed on the previously selected molecules to evaluate their binding affinities towards the BRAF protein. The results revealed that, on average, the generated ligands exhibited a binding affinity of −8.03 ± 1.12 kcal/mol. Notably, the ligand with the highest binding affinity achieved a remarkable value of −11.5 kcal/mol.

The molecular docking analysis was performed on the selected AI-generated compounds to evaluate their binding affinities toward the BRAF protein. The results revealed that the generated ligands exhibited an average binding affinity of −8.03 ± 1.12 kcal/mol, with the top-performing ligand achieving −11.5 kcal/mol—a value comparable to or exceeding FDA-approved BRAF inhibitors. For context, control docking of clinical inhibitors under identical conditions yielded scores of −7.4 kcal/mol (encorafenib), −11.0 kcal/mol (dabrafenib), −11.0 kcal/mol (regorafenib), and −10.9 kcal/mol (sorafenib). While these computational results suggest superior or equivalent target engagement for our top ligand (−11.5 kcal/mol), we acknowledge that *in vitro* validation (*e.g*., SPR or FP assays) is required to confirm these predictions.

During the selection process, four molecules with binding affinity values lower than −10.5 kcal/mol demonstrated exceptional drug-like properties, with QED values exceeding 8.2 and Chemprop predicted values greater than 7. The detailed predicted physicochemical properties of these four molecules, in comparison to known drugs, are summarized in Table 1. The selected generated ligands (Ligand 1 to Ligand 4) exhibit favorable drug-like properties, with molecular weights (MW) ranging from 381.47 to 410.55 g/mol, low numbers of rotatable bonds (5), and moderate lipophilicity (iLOGP: 3.6–4.18), suggesting good membrane permeability and oral bioavailability. Their topological polar surface area (TPSA: 49.74–70.59 Å^2^) and hydrogen bonding profiles further support their high gastrointestinal (GI) absorption, unlike the known drugs (ENCORAFENIB, DABRAFENIB, SORAFENIB, REGORAFENIB), which show low GI absorption due to higher TPSA values and molecular weights. However, the ligands are predicted to be P-glycoprotein (Pgp) substrates, which may impact their efflux and bioavailability. Their cytochrome P450 (CYP) inhibition profiles vary, with Ligand 1 and Ligand 3 showing selective inhibition patterns, while TXV exhibits broad CYP inhibition. Notably, their synthetic accessibility scores (3.3–3.71) suggest they are feasible to synthesize. Overall, these molecules demonstrate promising physicochemical properties for further development as drug candidates, while warranting early ADMET risk de-risking.

**Table 1:** Comparative analysis of predicted physicochemical and pharmacokinetic properties for four candidate molecules relative to established drug compounds.

Molecule	1	2	3	4	ENCORAFENIB	DABRAFENIB	SORAFENIB
MW	409.52	410.55	399.46	381.47	540.01	519.56	464.82
Num. heavy atoms	30	30	29	28	36	35	32
Num. rotatable bonds	5	5	5	5	11	6	9
Num. H-bond acceptors	3	4	5	3	8	8	7
Num. H-bond donors	1	1	1	1	3	2	3
TPSA	63.57	49.74	70.59	63.57	148.51	147.48	92.35
iLOGP	3.82	4.18	3.83	3.6	2.95	3.08	3.42
GI absorption	High	High	High	High	Low	Low	Low
BBB permeant	Yes	Yes	Yes	Yes	No	No	No
Pgp substrate	Yes	Yes	Yes	Yes	Yes	No	No
CYP1A2 inhibitor	No	No	No	No	No	No	Yes
CYP2C9 inhibitor	Yes	No	Yes	No	No	Yes	Yes
CYP2D6 inhibitor	Yes	Yes	Yes	Yes	No	No	Yes
Lipinski Num. violations	0	0	0	0	2	1	0
Bioavailability score	0.55	0.55	0.55	0.55	0.17	0.55	0.55
Synthetic accessibility	3.53	3.71	3.3	3.31	4.35	4.05	2.87

To anticipate off-target pharmacology arising from the scaffold novelty, we performed a Similarity Ensemble Approach (SEA) (Keiser et al., 2007) analysis across both GPCRs, kinases, and epigenetic target families (full results in Data S4). Consistent with our design objective, BRAF was a top-ranked target for three ligands (high Z-scores and sub-extreme *p*-values), and RAF1 was prominently ranked for Ligand 2, supporting the intended target engagement. Importantly, SEA also flagged potential off-targets, including: (1) high-affinity binding to serotonergic GPCRs (5-HT1B/1D) for Ligand 1 and Ligand 4 (*p* < 0.001, Z > 3.0), suggesting potential vasoactive effects such as peripheral vasoconstriction upon receptor modulation; (2) variable-confidence predictions across multiple non-RAF kinases (ABL1, BTK, BMX, SYK, AKT1) that may confer immunomodulatory or myelosuppressive risks if cellular activity is confirmed; and (3) recurrent epigenetic modulator hits (BRD4/BRPF1 bromodomains and PRMT6/CARM1/EHMT1/2 methyltransferases) indicating possible transcriptional dysregulation at elevated exposures, all requiring experimental validation due to the similarity-based nature of SEA predictions. Given the similarity-based nature of SEA, these predictions require experimental confirmation.

**Figure 4:**
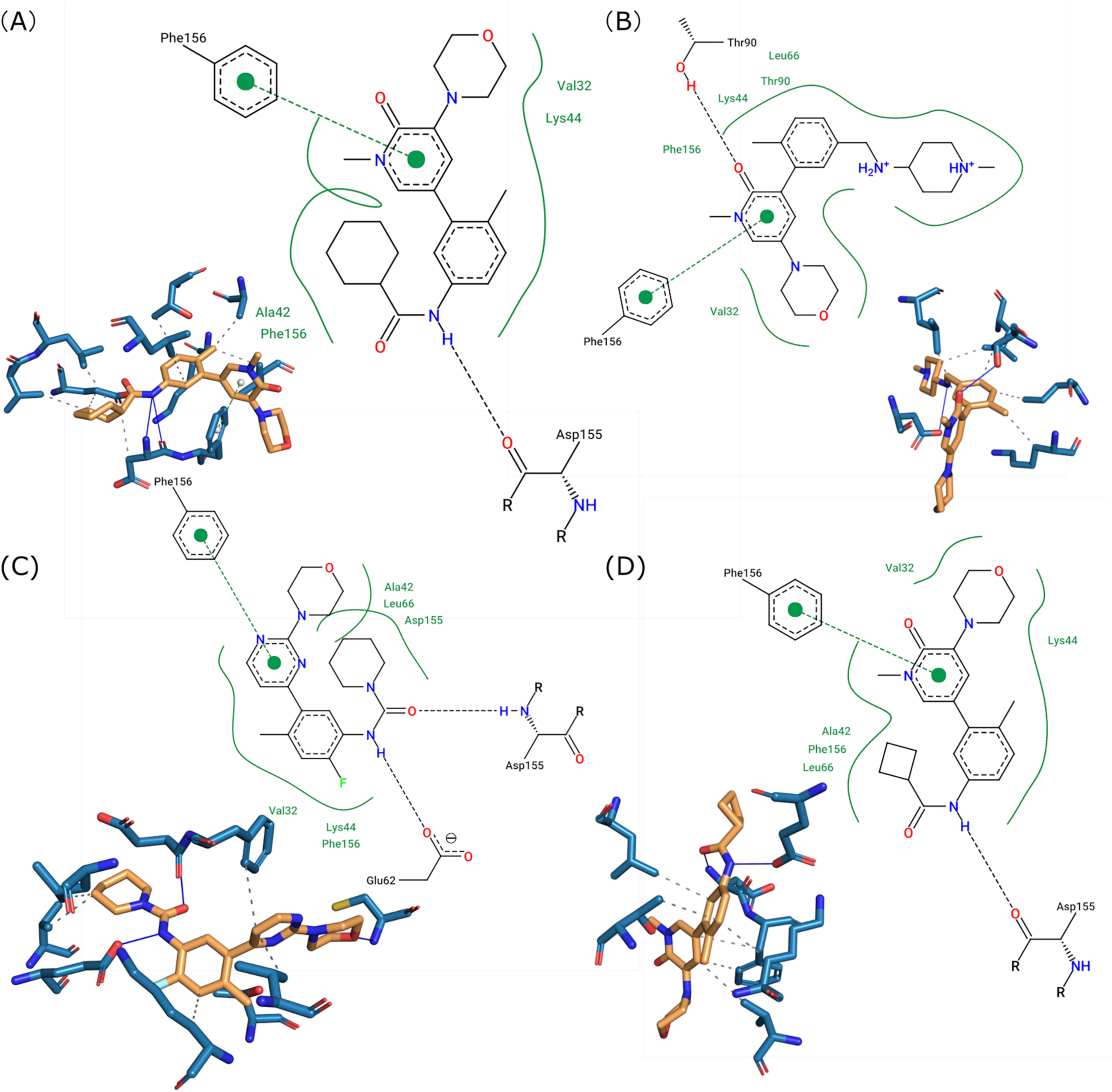
Three-dimensional (3D) and two-dimensional (2D) visualizations of the selected four docked ligands interacting with the BRAF V600E mutant. In the 3D visualization, hydrogen bonds are depicted as solid blue lines, hydrophobic interactions as dashed gray lines, and π-stacking interactions as dashed green lines. In the 2D visualization, hydrogen bonds, hydrophobic interactions, and π-stacking interactions are represented by black dashed lines, solid green lines, and dashed green lines, respectively.

These ligands were subjected to further investigation to elucidate their interactions with the BRAF protein. Figure 4 illustrates the molecular interactions between the ligands and BRAF, highlighting the key amino acid residues involved in ligand binding. The binding affinity values for the four ligands were determined to be −11.5, −11.1, −11.1, and −10.6 kcal/mol, indicative of robust interactions with the protein. The ligand with the highest binding affinity (−11.5 kcal/mol) engaged in multiple interactions, including hydrogen bonds with Asp155 and Glu62, as well as hydrophobic contacts with Lys44, Thr90, and Val32. Similarly, the other ligands, despite slightly lower affinities, consistently interacted with critical residues such as Lys44, Thr90, and Phe156 through hydrophobic interactions, and with Asp155 and Glu62 *via* hydrogen bonding. These findings underscore the significance of these residues in stabilizing the ligand-receptor complex and suggest a positive correlation between the number of interactions and binding affinity. The consistent involvement of Lys44, Thr90, Asp155, and Glu62 across all ligands highlights their pivotal role in BRAF-ligand binding, providing valuable insights for structure-based drug design.

### Molecular dynamics simulation

To assess the stability and intermolecular interaction dynamics over time, MD simulations were conducted on the four docked complexes. The simulations produced a range of analytical metrics, including root-mean-square deviation (RMSD), root-mean-square fluctuation (RMSF), the number of hydrogen bonds, and the radius of gyration (Rg). These parameters were systematically analyzed to evaluate the structural integrity, conformational flexibility, and interaction patterns of the complexes throughout the simulation trajectory. To ensure reproducibility, all simulations were performed in duplicate with different initial random velocities.

#### Root mean square deviation analysis

**Figure 5:**
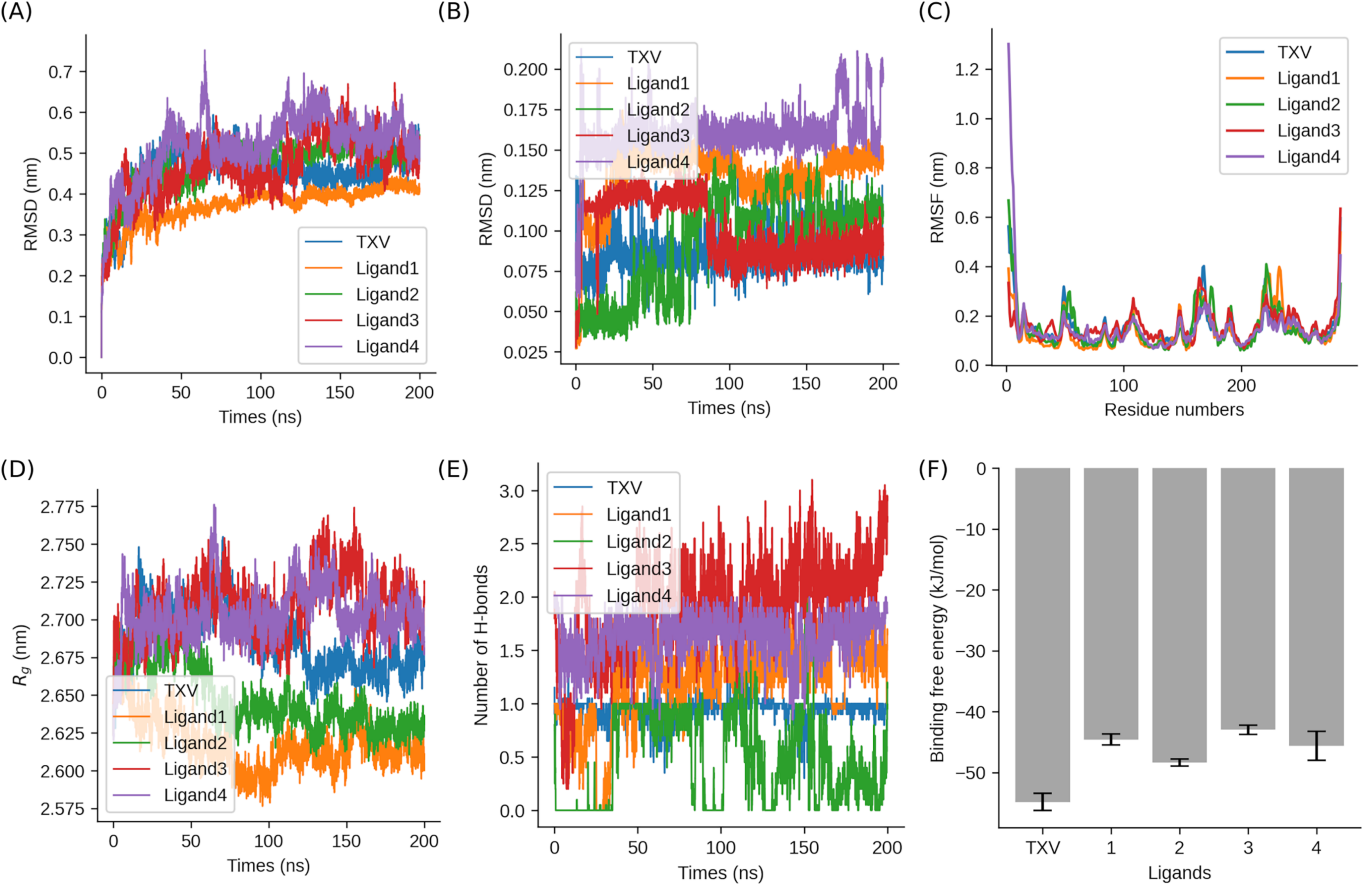
Analysis of MD trajectories from simulations of BRAF complex. In each case, MD simulation of 200 ns was performed. (A) RMSD of the protein backbone in complex with the four selected ligands and the referenced TXV. (B) RMSD of the five ligands. (C) Root-mean-square fluctuation (RMSF) analysis was performed on the C α atoms of residues. (D) Radius of gyration (Rg) of the BRAF complex structure. (E) Protein-Ligand Hydrogen Bonds Analysis. The number of hydrogen bonds between BRAF and the selected ligands is depicted, with a 50-ps running average plotted for improved clarity. (F) Binding free energy of the five ligands with BRAF.

The root-mean-square deviation (RMSD) was computed for the backbone atoms of both the protein and the ligand in each complex to evaluate system stability and quantify conformational changes in the ligands and the protein. The RMSD profiles for all simulated systems are illustrated in Fig. 5A.

The analysis demonstrated that the RMSD values of the proteins across all systems ranged between approximately 0.35 and 0.55 nm. After approximately 50 ns of simulation, the RMSD values for all systems converged to a plateau with minimal fluctuations, indicating sustained protein stability throughout the simulation. This stability suggests that the selected compounds were effectively stabilized and well-accommodated within the protein’s active site. Among the complexes, the protein in the Ligand 4 complex exhibited the highest overall RMSD, with discernible fluctuations, albeit within an acceptable range. Notably, in the two independent replicates of the Ligand 1 complex, the RMSD values showed the largest variation (0.37 ± 0.04 nm *vs*. 0.59 ± 0.11 nm), despite both trajectories ultimately converging (Fig. S1). This observation highlights the importance of conducting multiple replicates to assess conformational sampling reproducibility, even when stability is achieved.

To rigorously assess ligand stability, the RMSD of each ligand relative to its initial conformation was calculated for both independent simulation replicates (Fig. 5B). The primary simulations yielded mean RMSD values of 0.086 nm (TXV), 0.134 nm (Ligand 1), 0.091 nm (Ligand 2), 0.101 nm (Ligand 3), and 0.161 nm (Ligand 4), with standard deviations (SD) between 0.014–0.031 nm. Duplicate simulations confirmed these trends, showing comparable mean RMSDs (Fig. S1): 0.086 nm (TXV), 0.179 nm (Ligand 1), 0.054 nm (Ligand 2), 0.139 nm (Ligand 3), and 0.169 nm (Ligand 4) (SD: 0.009–0.024 nm).

Ligand 4 exhibited the highest mean RMSD values (0.161 ± 0.017 nm in the primary run; 0.169 ± 0.022 nm in the duplicate), confirming its pronounced flexibility within the binding pocket. Conversely, Ligand 2 displayed the greatest structural rigidity, with exceptionally low RMSD values in both simulations (0.091 ± 0.031 nm *vs*. 0.054 ± 0.010 nm), suggesting a tightly constrained binding mode. Ligand 3 exhibited intermediate stability, though with notable deviation between replicates (0.101 ± 0.019 nm *vs*. 0.139 ± 0.015 nm), implying mild conformational plasticity.

Most notably, Ligand 1 exhibited significant inter-replicate variability in RMSD measurements (0.134 ± 0.020 nm *vs*. 0.179 ± 0.024 nm), demonstrating substantially greater fluctuation than other tested compounds. Subsequent hydrogen bonding analysis revealed parallel inconsistencies in inter-replicate observations, potentially accounting for the observed RMSD discrepancies. Crucially, all measured values fell within established biophysical tolerance thresholds (<0.2 nm), suggesting these variations likely reflect inherent stochastic ligand dynamics rather than systemic methodological artifacts.

#### Root mean square fluctuation analysis

The root mean square fluctuation (RMSF) is a widely employed metric for evaluating the dynamic behavior of amino acid residues within a protein structure. In this study, the RMSF of Cα atoms was calculated for all protein-ligand complexes, with average values ranging from 0.1 to 0.4 nm, as illustrated in Fig. 5C (excluding the terminal regions). The relatively low RMSF values observed across the complexes indicate that the protein-ligand systems adopt stable conformations while retaining a moderate degree of flexibility, which is crucial for their functional dynamics.

The RMSF of Cα atoms was calculated across all protein-ligand complexes to evaluate residue-specific flexibility. Primary simulations (five independent replicates) exhibited consistent mean RMSF values (0.14–0.16 nm, SD: 0.06–0.13 nm, Fig. 5C), while duplicate runs showed slightly elevated fluctuations (mean: 0.15–0.21 nm, SD: 0.07–0.12 nm), suggesting minor variability in conformational sampling. The overall low RMSF values (majority < 0.3 nm) indicate stable protein-ligand conformations with moderate functional flexibility, corroborated by high inter-replicate correlation (Pearson r = 0.56–0.80, *p* < 0.001).

A detailed analysis of the RMSF profiles revealed that, in addition to the anticipated higher fluctuations at the N- and C-terminal regions, several other amino acid residues exhibited significant RMSF variations. A comparison with the crystal structure of the protein demonstrated that two regions with elevated B-factor values—located near residue A50 and between residues D148 and S168—also displayed pronounced RMSF fluctuations in all five MD simulations. This alignment between high B-factor regions in the crystal structure and elevated RMSF values in the MD simulations highlights the inherent dynamic nature of these regions.

Notably, the RMSF profiles revealed distinct differences among the complexes. For instance, the Ligand 3 complex exhibited a pronounced fluctuation near residue D40, whereas the other complexes demonstrated relatively stable behavior in this region. These variations in RMSF values provide valuable insights into the specific interactions and conformational dynamics induced by different ligands, which may influence their binding stability and functional efficacy.

#### Radius of gyration analysis

The compactness and structural stability of the protein-ligand complexes were assessed using the radius of gyration (Rg), a parameter defined as the mass-weighted root mean square distance of the atomic distribution from their mutual center of mass (Lobanov, Bogatyreva & Galzitskaya, 2008). A lower Rg value is indicative of a more compact and stable protein conformation.

Rg analysis indicated consistent structural compactness across complexes (primary: 2.62–2.71 nm; duplicate: 2.71–2.76 nm). Ligand 1 yielded the most compact assemblies (2.618 ± 0.019 nm), Ligand 3 the least (2.709 ± 0.021 nm), and Ligand 2 intermediate configurations (2.648 ± 0.022 nm). Notably, duplicate simulations revealed system-dependent stability patterns. Ligand 1 showed significant conformational variation (ΔRg = 0.138 nm), suggesting binding-induced metastability (Fig. S2). In contrast, Ligand 3 complexes maintained consistent Rg values (ΔRg < 0.025 nm between replicates), indicating robust structural reproducibility. Ligand 2’s duplicate (2.708 ± 0.020 nm) showed improved compactness relative to its primary run. The tight Rg distributions (SD < 0.04 nm) confirm all systems maintained stable folds. However, Ligand 1’s greater variability highlights its dynamic binding nature, whereas Ligand 3’s consistently higher Rg values (mean > 2.70 nm) reflect its distinct binding mode.

#### Hydrogen bonds and binding free energy

The 200-ns molecular dynamics simulations provided mechanistic insights into ligand-receptor recognition patterns. TXV demonstrated exceptional binding stability, maintaining the highest average hydrogen bond count (2.49 ± 0.00 across replicates) among all tested compounds. This observation correlates with its superior binding affinity (ΔG = −56.94 ± 2.77 kcal/mol in primary runs; −56.32 ± 2.84 kcal/mol in duplicates), suggesting an optimized polar interaction network despite its relatively higher molecular mass. The remarkable consistency between independent simulation replicates (ΔΔG < 0.62 kcal/mol) further validates the robustness of these interactions.

Ligand 3 displayed notable variability: while its primary run achieved near-TXV affinity (−55.90 ± 3.12 kcal/mol) with 1.92 hydrogen bonds, the duplicate showed a 52% drop in hydrogen bonds (0.91) and weaker binding (−49.17 ± 2.46 kcal/mol), attributed to GLU62 displacement from the active center. This highlights how subtle conformational changes can disrupt key polar interactions. Ligand 4 maintained moderate hydrogen bonding (1.63–1.64) and consistent affinity (−53.18 to −50.90 kcal/mol), with tight SEM (<0.30 kcal/mol) suggesting reproducible binding. In contrast, Ligand 1’s duplicate exhibited enhanced hydrogen bonding (1.70 *vs*. 1.21) and improved affinity (−53.55 *vs*. −49.71 kcal/mol), implying alternate binding modes. Ligand 2, with the weakest interactions (0.42–0.47 hydrogen bonds), consistently had the poorest affinity (−48.35 to −47.75 kcal/mol), reflecting its reliance on transient polar contacts.

#### Stability of binding modes

**Figure 6:**
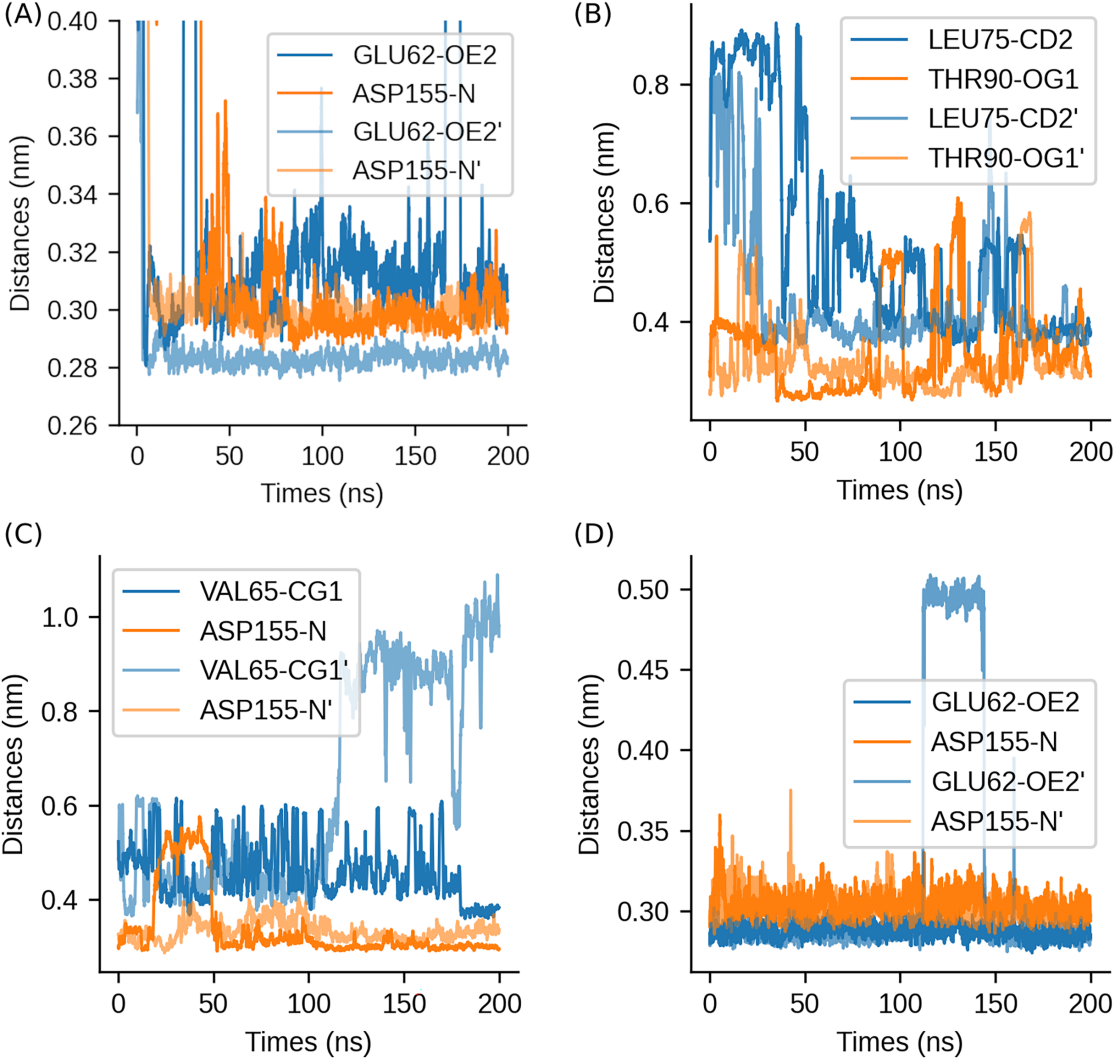
Evolution of key ligand-residue distances during 200 ns MD simulations. (A) Ligand 1 complex, (B) Ligand 2 complex, (C) Ligand 3 complex, (D) Ligand 4 complex. Critical interacting residues are labeled for each replicate simulation, with prime notation (′) distinguishing independent runs (*e.g*., ASP155-N *vs*. ASP155-N′).

To validate the stability of ligand binding, we monitored the evolution of key distances between ligand functional groups and active site residues across all systems (Fig. 6). These distances were selected based on their critical roles in hydrogen bonding and hydrophobic interactions, as identified in prior analyses.

The binding mode of Ligand 1 complex exhibited unique characteristics in our molecular dynamics simulations. Notably, the interaction between ASP155-OE2 and the ligand demonstrated remarkable stability across both replicate simulations, as evidenced by nearly superimposable trajectories (Fig. 6). In contrast, the distance between GLU62-OE2 and the ligand displayed significant variability between replicates-one replicate maintained a stable distance of 0.28 nm, while the other showed pronounced fluctuations around 0.31 nm.

The other three ligand complexes exhibited significantly higher consistency in their duplicate simulations, with well-maintained structural stability throughout the simulation period. Hydrogen bond interactions in these complexes remained particularly stable, although hydrophobic interactions demonstrated greater positional variability (Fig. 6). Ligand 2 showed comparatively weaker binding stabilization, with THR90 forming a hydrogen bond during only 50% of the simulation time and displaying fluctuating hydrophobic interactions with LEU75-CD2 (0.40–0.55 nm). In contrast, Ligand 4 achieved exceptional binding stability in both runs, forming persistent hydrogen bonds with GLU62 (0.29 ± 0.015 nm) and ASP155 (0.30 ± 0.020 nm). These stable interactions, coupled with a high calculated binding free energy (−53.18 kcal/mol, primary run), account for the highly convergent RMSD profile observed for this ligand-receptor complex.

## Conclusions

In conclusion, this study effectively utilized the REINVENT 4 framework, integrating reinforcement learning and transfer learning, to design novel BRAF V600E inhibitors with improved drug-likeness and binding affinity. The AI-driven approach not only improved molecular diversity but also generated compounds with favorable binding affinities, outperforming or matching existing FDA-approved inhibitors in molecular docking analyses. These advancements, validated through predictive modeling, docking, and molecular dynamics simulations, showcase the potential of AI to expedite the development of targeted cancer therapies.

However, several limitations must be addressed. First, while molecular docking and dynamics simulations provided valuable insights into binding affinity and stability against BRAF V600E, *in vitro* experimental validation is crucial to confirm the predicted biological activity. Second, the present study did not assess the selectivity of the generated molecules against wild-type BRAF or other kinases in the wider kinome, which is essential to minimize off-target effects and will be systematically investigated in follow-up work. Additionally, although the generated compounds exhibited promising drug-likeness, further studies on their pharmacokinetics, such as absorption, distribution, metabolism, excretion, and toxicity (ADMET) properties, are needed to fully assess their clinical viability. Finally, while the integration of RL and TL improved the quality of generated compounds, further optimization of the learning models could enhance their predictive power and generalizability to other therapeutic targets. Future work will therefore focus on experimental validation, comprehensive kinome-wide selectivity profiling, and expanding the scope of AI applications for drug discovery.

## Supplemental Information

10.7717/peerj.20541/supp-1 Supplemental Information 1 Training dataset for transfer learning. A dataset of potential BRAF V600E inhibitors was sourced from the ChEMBL database, with compounds having a pIC50 greater than 7.2 being retained. A total of 1,671 compounds were obtained.

10.7717/peerj.20541/supp-2 Supplemental Information 2 Parameters for reinforcement learning (RL), transfer learning(TL), chemprop training, and docking. Parameters for first stage RL, TL, second stage RL, chemprop model training, and docking.

10.7717/peerj.20541/supp-3 Supplemental Information 3 Dataset of generated compounds including SMILES strings and associated properties. Dataset generated from the final stage of the *de novo* compound generation process.

10.7717/peerj.20541/supp-4 Supplemental Information 4 Similarity Ensemble Approach (SEA) analysis of selected molecules. SEA was performed to evaluate potential off-target pharmacology. BRAF emerged as a top-ranked target for three ligands (high Z-scores and sub-extreme p-values), RAF1 showed prominent ranking for Ligand 2.

10.7717/peerj.20541/supp-5 Supplemental Information 5 Convergence analysis of duplicate molecular dynamics simulations. (A-D) Protein backbone RMSD (upper) and ligand heavy-atom RMSD (lower) trajectories for Systems 1-4. Black and red lines indicate two independent replicates initiated with different random velocities.

10.7717/peerj.20541/supp-6 Supplemental Information 6 Radius of gyration (Rg) analysis for four molecular dynamics (MD) systems. (A–D) Trajectories from two independent replicate simulations are overlaid for each system from Ligand 1 to Ligand 4 complex (replicate 1: red; replicate 2: black), demonstrating the convergence of protein compactness dynamics across runs. Rg values (nm) were calculated for all backbone atoms at 100-ps intervals over the full simulation timecourse.

## Additional Information and Declarations

### Data Availability

The following information was supplied regarding data availability:

The data is available in the Supplemental Files.

All the relevant structure, topology, and trajectory files are available at ScienceDB: Zuokun Lu (2025). Molecular Dynamics Simulation Data of Five BRAF V600E Inhibitor Complexes: AI-Designed Small Molecules in Oncology. V1. Science Data Bank. https://doi.org/10.57760/sciencedb.26031.
