# Supplementary material for: AI-driven de novo design of BRAF inhibitors with enhanced binding affinity and optimized drug-likeness
Source: PeerJ. 2026 Jan 2;14:e20541. doi: 10.7717/peerj.20541 (PMC12767490; doi:10.7717/peerj.20541)
Supplement: Supplemental Information 2 — Parameters for first stage RL, TL, second stage RL, chemprop model training, and docking. [file peerj-14-20541-s002.docx]

# First RL pramenters

stage1_parameters=f"""

run_type = "staged_learning"

device = "cuda:0"

tb_logdir = "tb_stage1"

json_out_config = "_stage1.json"

[parameters]

prior_file = "{prior_filename}"

agent_file = "{agent_filename}"

summary_csv_prefix = "stage1"

batch_size = 100

use_checkpoint = false

[learning_strategy]

type = "dap"

sigma = 128

rate = 0.0001

[[stage]]

max_score = 1.0

max_steps = 300

chkpt_file = "{stage1_checkpoint}"

scoring_function.type = "custom_product"

[stage.scoring]

type = "geometric_mean"

[[stage.scoring.component]]

[stage.scoring.component.custom_alerts]

[[stage.scoring.component.custom_alerts.endpoint]]

name = "Alerts"

params.smarts = [

"[*;r8]",

"[*;r9]",

"[*;r10]",

"[*;r11]",

"[*;r12]",

"[*;r13]",

"[*;r14]",

"[*;r15]",

"[*;r16]",

"[*;r17]",

"[#8][#8]",

"[#6;+]",

"[#16][#16]",

"[#7;!n][S;!$(S(=O)=O)]",

"[#7;!n][#7;!n]",

"C#C",

"C(=[O,S])[O,S]",

"[#7;!n][C;!$(C(=[O,N])[N,O])][#16;!s]",

"[#7;!n][C;!$(C(=[O,N])[N,O])][#7;!n]",

"[#7;!n][C;!$(C(=[O,N])[N,O])][#8;!o]",

"[#8;!o][C;!$(C(=[O,N])[N,O])][#16;!s]",

"[#8;!o][C;!$(C(=[O,N])[N,O])][#8;!o]",

"[#16;!s][C;!$(C(=[O,N])[N,O])][#16;!s]"

]

[[stage.scoring.component]]

[stage.scoring.component.QED]

[[stage.scoring.component.QED.endpoint]]

name = "QED"

weight = 0.6

[[stage.scoring.component]]

[stage.scoring.component.NumAtomStereoCenters]

[[stage.scoring.component.NumAtomStereoCenters.endpoint]]

name = "Stereo"

weight = 0.4

transform.type = "left_step"

transform.low = 0

"""

# Transfer learning parameter

TL_parameters = f"""

run_type = "transfer_learning"

device = "cuda:0"

tb_logdir = "tb_TL"

[parameters]

num_epochs = 50

save_every_n_epochs = 2

batch_size = 100

sample_batch_size = 10000

input_model_file = "{stage1_checkpoint}"

output_model_file = "TL_reinvent.model"

smiles_file = "{TL_train_filename}"

validation_smiles_file = "{TL_validation_filename}"

standardize_smiles = true

randomize_smiles = true

randomize_all_smiles = false

internal_diversity = true

"""

# Stage 2 RL parameter

TL_model_filename = os.path.join(wd, "TL_reinvent.model.30.chkpt")

stage2_parameters = re.sub("stage1", f"stage2", stage1_parameters)

stage2_parameters = re.sub("agent_file.*\n", f'agent_file = "{TL_model_filename}"\n', stage2_parameters)

stage2_parameters = re.sub("max_steps.*\n", f'max_steps = 1000\n', stage2_parameters)

pred_model_parameters = f"""

[[stage.scoring.component]]

[stage.scoring.component.ChemProp]

[[stage.scoring.component.ChemProp.endpoint]]

name = "ChemProp"

weight = 0.6

params.checkpoint_dir = "{chemprop_path}"

params.rdkit_2d_normalized = true

transform.type = "sigmoid"

transform.high = 10

transform.low = 4

transform.k = 0.4

"""

df_parameters = """

[diversity_filter]

type = "IdenticalMurckoScaffold"

bucket_size = 10

minscore = 0.7

"""

inception_parameters = """

[inception]

smiles_file = "" # no seed SMILES

memory_size = 50

sample_size = 10

"""

full_stage2_parameters = stage2_parameters + pred_model_parameters + df_parameters + inception_parameters

stage2_config_filename = "stage2.toml"

# Chemprop command parameters

python /home/luzuokun/anaconda3/envs/reinvent4/bin/chemprop_train --data_path BRAF_smiles_pIC50.csv --dataset_type regression --save_dir exp1/ --split_type cv --num_folds 5 --features_generator rdkit_2d_normalized –no_features_scaling

# Docking parameters

Autodock vina 1.1.2 was used in DockStream, with the following parameters:

# specify the target preparation JSON file as a dictionary and write it out

tp_dict = {

"target_preparation":

{

"header": { # general settings

"logging": { # logging settings (e.g. which file to write to)

"logfile": log_file_target_prep

}

},

"input_path": apo_1UYD_path, # this should be an absolute path

"fixer": { # based on "PDBFixer"; tries to fix common problems with PDB files

"enabled": True,

"standardize": True, # enables standardization of residues

"remove_heterogens": True, # remove hetero-entries

"fix_missing_heavy_atoms": True, # if possible, fix missing heavy atoms

"fix_missing_hydrogens": True, # add hydrogens, which are usually not present in PDB files

"fix_missing_loops": False, # add missing loops; CAUTION: the result is usually not sufficient

"add_water_box": False, # if you want to put the receptor into a box of water molecules

"fixed_pdb_path": fixed_pdb_path # if specified and not "None", the fixed PDB file will be stored here

},

"runs": [ # "runs" holds a list of backend runs; at least one is required

{

"backend": "AutoDockVina", # one of the backends supported ("AutoDockVina", "OpenEye", ...)

"output": {

"receptor_path": adv_receptor_path # the generated receptor file will be saved to this location

},

"parameters": {

"pH": 7.4, # sets the protonation states (NOT used in Vina)

"extract_box": { # in order to extract the coordinates of the pocket (see text)

"reference_ligand_path": reference_ligand_path, # path to the reference ligand

"reference_ligand_format": "PDB" # format of the reference ligand

}

}}]}}

with open(target_prep_path, 'w') as f:

json.dump(tp_dict, f, indent=" ")

# specify the embedding and docking JSON file as a dictionary and write it out

ed_dict = {

"docking": {

"header": { # general settings

"logging": { # logging settings (e.g. which file to write to)

"logfile": log_file_docking

}

},

"ligand_preparation": { # the ligand preparation part, defines how to build the pool

"embedding_pools": [

{

"pool_id": "RDkit_pool", # here, we only have one pool

"type": "RDkit",

"parameters": {

"removeHs": False,

"coordinate_generation": {

"method": "UFF",

"maximum_iterations": 300

}

},

"input": {

"standardize_smiles": False,

"type": "smi",

"input_path": smiles_path

},

"output": { # the conformers can be written to a file, but "output" is

# not required as the ligands are forwarded internally

"conformer_path": ligands_conformers_path,

"format": "sdf"

}

}

]

},

"docking_runs": [

{

"backend": "AutoDockVina",

"run_id": "AutoDockVina",

"input_pools": ["RDkit_pool"],

"parameters": {

"binary_location": vina_binary_location, # absolute path to the folder, where the "vina" binary

# can be found

"parallelization": {

"number_cores": 16

},

"seed": 42, # use this "seed" to generate reproducible results; if

# varied, slightly different results will be produced

"receptor_pdbqt_path": [adv_receptor_path], # paths to the receptor files

"number_poses": 2, # number of poses to be generated

"search_space": { # search space (cavity definition); see text

"--center_x": 25.9,

"--center_y": 110.95,

"--center_z": 16.6,

"--size_x": 16,

"--size_y": 12,

"--size_z": 15

}

},

"output": {

"poses": { "poses_path": ligands_docked_path },

"scores": { "scores_path": ligands_scores_path }

}}]}}

with open(docking_path, 'w') as f:

json.dump(ed_dict, f, indent=2)

# print out path to generated JSON

print(docking_path)
