# Supplementary figures and images for: AI-driven de novo design of BRAF inhibitors with enhanced binding affinity and optimized drug-likeness
Source: PeerJ. 2026 Jan 2;14:e20541. doi: 10.7717/peerj.20541 (PMC12767490; doi:10.7717/peerj.20541)

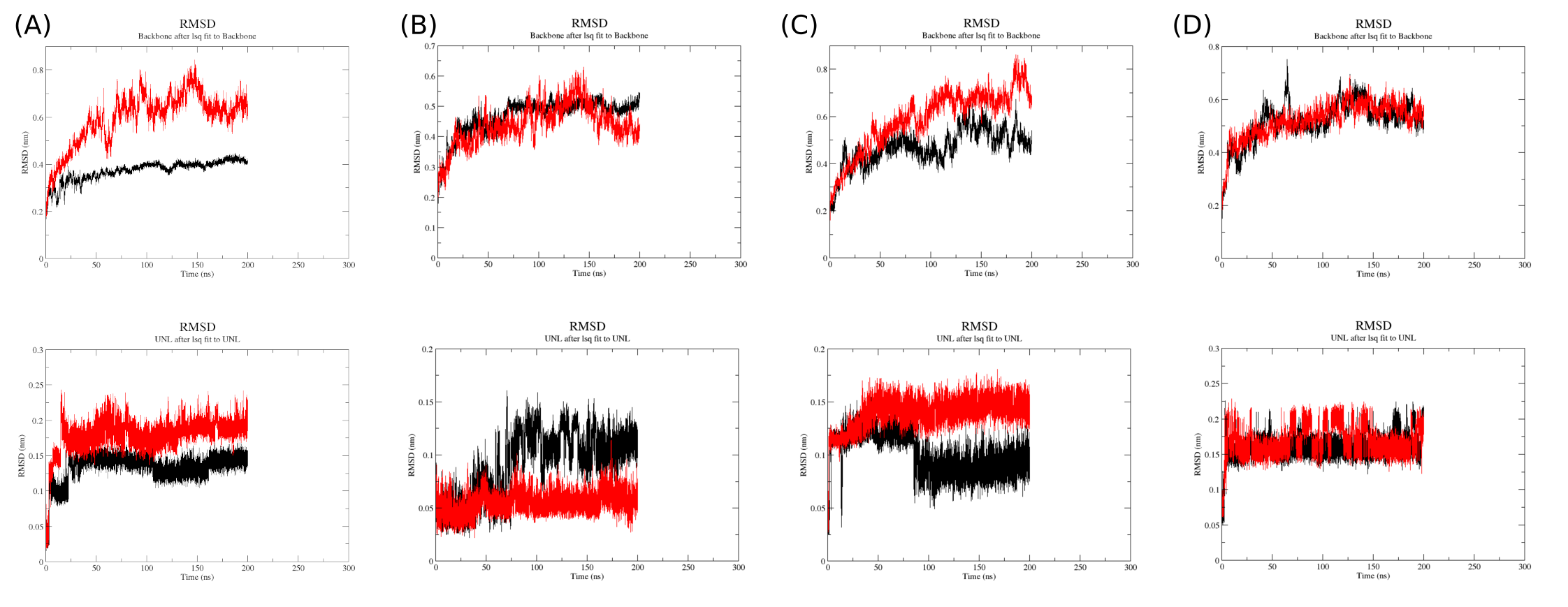

Supplement: Supplemental Information 5 — (A-D) Protein backbone RMSD (upper) and ligand heavy-atom RMSD (lower) trajectories for Systems 1-4. Black and red lines indicate two independent replicates initiated with different random velocities. [file peerj-14-20541-s005.png]

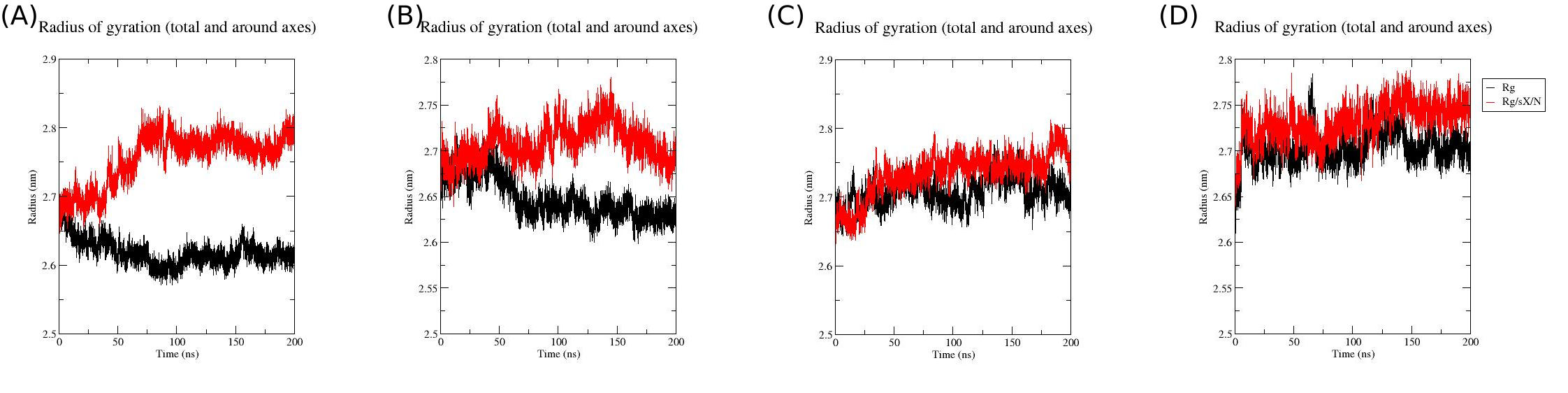

Supplement: Supplemental Information 6 — (A–D) Trajectories from two independent replicate simulations are overlaid for each system from Ligand 1 to Ligand 4 complex (replicate 1: red; replicate 2: black), demonstrating the convergence of protein compactness dynamics across runs. Rg values (nm) were calculated for all backbone atoms at 100-ps intervals over the full simulation timecourse. [file peerj-14-20541-s006.png]
